# The novel outer membrane protein from OprD/Occ family is associated with hypervirulence of carbapenem resistant Acinetobacter baumannii ST2/KL22

**DOI:** 10.1080/21505594.2020.1856560

**Published:** 2020-12-29

**Authors:** Mingxi Hua, Jingyuan Liu, Pengcheng Du, Xinzhe Liu, Min Li, Huizhu Wang, Chen Chen, Xinmin Xu, Yu Jiang, Yajie Wang, Hui Zeng, Ang Li

**Affiliations:** a Institute of Infectious Diseases, Beijing Ditan Hospital, Capital Medical University, Beijing; b Department of Critical Care Medicine, Beijing Ditan Hospital, Capital Medical University, Beijing; c Beijing Key Laboratory of Emerging Infectious Diseases, Beijing; d Clinical Laboratory, Beijing Ditan Hospital, Capital Medical University, Beijing, China; e Department of Stomatology, Beijing Children’s Hospital, Capital Medical University, Beijing

**Keywords:** *Acinetobacter baumanii*, sequence types 2, KL22, OrpD, antibiotic resistance

## Abstract

*Acinetobacter baumannii* has become a major healthcare threat that causes nosocomial infections, especially in critically ill patients. The spread of carbapenem-resistant *A. baumannii* (CRAB) strains has long been a clinical concern. It is important to study the epidemiology and virulence characteristics of different CRAB isolates in order to tailor infection prevention and antibiotic prescribing. In this study, a total of 71 CRAB isolates were collected in the hospital, and clinical characteristics of infections were analyzed. The genomic characteristics and phylogenetic relationships were elucidated based on genome sequencing and analysis. The isolates were assigned to three sequence types (STs, Pasteur) and nine capsular polysaccharide (KL) types, among which ST2/KL22 was the most prevalent CRAB in the hospital. Even though all the ST2/KL22 isolates contained the same reported virulence genes, one specific clade of ST2/KL22 showed more pathogenic in mouse infection model. Complete genomic analysis revealed differences at the *oprD* locus between the low- and high-virulent isolates. More specifically, a premature stop codon in the low-virulence strains resulted in truncated OprD expression. By evaluating pathogenicity in C57BL/6 J mice, knock-out of *oprD* in high-virulent isolate resulted in virulence attenuation, and complementing the avirulent strain with full-length *oprD* from high-virulent isolate enhanced virulence of the former. The *oprD* gene may be associated with the enhanced virulence of the specific ST2/KL22 clone, which provides a potential molecular marker for screening the hypervirulent *A. baumannii* strains.

## Introduction

*Acinetobacter baumannii* has become a major health threat, causing nosocomial infections such as ventilator-associated pneumonia and bacteremia, especially in intensive care units (ICUs) [1] (https://www.who.int/medicines/publications/global-priority-list-antibiotic-resistant-bacteria/en/). Genome analysis indicates that *A. baumannii* epidemics are caused by a limited number of strains that belong to the international clonal lineages (IC) I, II, or III. In particular, IC II strains represented by sequence type 2 (ST2, Pasteur scheme) are the predominant cause of outbreaks [2]. These infections have become exceedingly difficult to treat due to high levels of antibiotic resistance [3]. In 2019, the Centers for Disease Control and Prevention listed carbapenem-resistant *A. baumannii* (CRAB) as one of the top urgent threats (https://www.cdc.gov/drugresistance/biggest-threats).More than 60% of *A. baumannii* pneumonia cases worldwide are caused by CRAB [4–6].

Beyond drug resistance, fundamental virulence mechanisms enable *A. baumannii* to successfully thrive in the host and health-care environment [7]. Surface-exposed structures play a crucial role in the survival, drug resistance, and pathogenicity of *A. baumannii* [8,9]. Csu pili and biofilm-associated proteins (BAPs) and BAP-like proteins (BLPs) greatly contribute to the formation and maintenance of biofilms, which enable the persistence of *A. baumanii* under environmental insult and contribute to intrinsic antibiotic resistance [10]. Other surface proteins like capsular polysaccharides (CPS), lipopolysaccharides (LPS), and the type VI secretion system play major roles in the pathogenicity of CRAB through interactions with the host and competitors [11,12]. However, strains of the same sequence types with the same virulence genes show different pathogenicity in *Galleria mellonella* models, which implies undiscovered virulence factors at play during the early stages of infection [5]. Therefore, tracking dominant strains of CRAB in the hospital environment and characterizing their specific virulence characteristics is crucial for the prevention and treatment of nonsocial infections.

In this study, we performed whole-genome sequencing of 71 CRAB isolates collected from a Chinese hospital to characterize the population structure and virulence characteristics of these isolates. Our results revealed a long-standing and enhanced virulence ST2/KL22 clone in the hospital. OrpD is a novel virulence factor associated with the pathogenicity of ST2/KL22 isolates.

## Methods

### Bacterial isolation and antimicrobial susceptibility testing

A total of 71 independent CRAB isolates were cultured from 70 patients and a stethoscope in different departments of Beijing Ditan Hospital from 2017 to 2019. The isolates were cultured on LB-Agar plates at 37°C for 18 hours (Oxoid, USA). Bacterial identification and *in vitro* antimicrobial susceptibility testing were performed using the BD Phoenix system (BD, USA) according to the 2017 Clinical and Laboratory Standards Institute (CLSI) Guidelines.

### Total DNA extraction and whole genome sequencing

The bacteria were cultured at 37°C for 16 hours in LB broth media, then total DNA was extracted using the Genomic DNA Kit (Qiagen, USA) according to the manufacturer’s instructions. Sequencing libraries were generated using the NEBNext® Ultra™ DNA Library Prep Kit for Illumina (NEB, USA), and the whole genome of the 71 CRAB strains was sequenced using the Illumina NovaSeq platform. The clean read data were obtained using fastQC [13].

Four isolates were randomly selected from the high and low-virulence clades for further complete genome sequencing. Long-reads were obtained using the Oxford Nanopore Technologies MinION platform. Then the complete genome sequences were assembled by combining highly accurate short-reads and the long-reads using Unicycler [14]. The assembled genomes were annotated using Prokka [15]. The core and pan-genome of the genomes were analyzed using Roary [16]. Antimicrobial resistance genes were identified by performing searches against the ResFinder database, and virulence genes were identified by performing searches against the Virulence Factor Database (VFDB) [17]. The gene clusters and types of capsular polysaccharide (KL) and lipooligosaccharide outer core (OCL) synthesis were identified using Kaptive software [18]. The subtypes of BAPs and BLPs were identified based on sequence alignment with the reference sequences from previous study [10]. The Illumina read data were deposited in the Sequencing Read Archive database SRP251775. The complete genomes of the four selected strains were deposited in the GenBank database (DT-Ab003: CP050916-CP050918, DT-Ab020:CP050911-CP050913, DT-Ab022: CP050907-CP050910, DT-Ab057: CP050904-CP050906).

### Phylogenetic analysis

The Illumina reads were first mapped to the complete genome sequence of *A. baumannii* strain KBN10P02143 (accession number: CP013924.1) using bowtie 2 software [19], and the results were filtered using Samtools [20]. Single nucleotide polymorphisms (SNPs) were identified using the iSNV-calling pipeline we constructed previously [21]. The variant sites conserved in all strains (core genome SNPs, cgSNPs) were retained, and the sequences of these sites in each strain were concatenated and employed for phylogenetic analysis using FastTree v2.1.10 with the maximum likelihood method [22].

### Multilocus sequence typing

Multilocus sequence typing (MLST) was performed based on both the Oxford and Pasteur schemes [23,24]. The genome sequences were compared with the nucleotide sequences of the housekeeping genes (*cpn60, gdhB, gltA, gpi, gdhB, recA*, and *rpoD* for Oxford scheme, and *cpn60, fusA, gltA, pyrG, recA, rplB, and rpoB* for Pasteur scheme) in the MLST database (https://pubmlst.org/organisms/acinetobacter-baumannii/), to determine the number of alleles and assign STs [25]. A clonal complex was defined as STs sharing alleles at five or six of seven loci [23,26] (http://eburst.mlst.net/).

### Virulence phenotype detection

Male C57BL/6 J mice (6–8 weeks old) were obtained from the Institute of Laboratory Animal Sciences, Chinese Academy of Medical Sciences (Beijing, China). All research animals were used in compliance with the guidelines of Institutional Animal Care and Use Committee of Capital Medical University.

For mouse infections, cultures of *A. baumannii* were adjusted to OD_600_ = 1.0 (1 × 10^9^ c.f.u/mL), and approximately 2 × 10^8^ c.f.u. were administered via intraperitoneal injection. The number of survival mouse was recorded once every two hours for 40 hours, intraperitoneal injection of saline was run as a control. For every single strain, a total of 30 mice were used for three replicate experiments (10 mice every single experiment), log-rank (Mantel-Cox) test was applied to test the significates of the mouse survival rate [27].

### Total RNA extraction and PCR amplification

The low virulent strain DT-Ab022 (LV) and high-virulent strain DT-Ab057 (HV) were cultured at 37°C for 16 hours in LB broth media, then total RNA was extracted using RNA extraction kit (Qiagen, Germany) according to the manufacturer’s directions. The RNA was subsequently treated with RNase-free DNase I (Qiagen, Germany) to degrade all genomic DNA. Complementarity DNA synthesis was performed using the PrimeScript RT reagent kit (Takara, Japan) according to the manufacturer’s directions. PCR was performed using the following primers (F1: CATTAACAACCGTGGCAATG;F2: CTATGACCGCTGGAAACCG; R: GTTAGTGCCGTGGTCTT), the PCR products were annealed at 55°C for 30 cycles using Thermocycler (Thermo Scientific, US).

### *Construction of* orpD *knock out and complement strains*

Primers for construction of *orpD* knock out and complement strains were designed based on the genome sequence of DT-Ab057 (HV), primers were listed in Table S1. Gene knockout was performed as previously described [28]. Briefly, primers *oprD*-UF/*oprD*-UR and *oprD*-DF/*oprD*-DR were used to amplify the upstream and downstream fragments of the *orpD* gene of DT-Ab057 (HV). Erythromycin resistance was used as the selective marker of the positive recombinant clones. Primers *oprD* EF/ER were used to amplify the erythromycin resistance gene *ermB*. The upstream fragments of the *orpD, ermB* gene, and downstream fragments of the *orpD* were assembled using Gibson assembly method, the three fragments were assembled using NEBuilder HiFi DNA Assembly Master Mix (NEB, US), then the recombinant fragment *orpD-*upstream-*ermB-orpD-*downstream was transferred to the DT-Ab057 (HV) competence cells by elec-transformation. The knock out strain was screened according to erythromycin resistance, and confirmed by PCR using the primers *oprD*-UF/*oprD*-DR.

Primers for *orpD* complement construction were designed based on the genome sequence of DT-Ab057 (HV). Primers *orpD* CF and *orpD* CR were used to amplify the complete *orpD* gene from DT-Ab057 (HV), and then the gene was cloned into the pMO13 plasmid at NotI/HindIII restriction site. The two fragments were assembled using NEBuilder HiFi DNA Assembly Master Mix to generate pMO13: *orpD*. The bases written in lowercase letters were the overlapping region with the pMO13 plasmid. The pMO13: *orpD* plasmid was transformed into *E. coli* TOP10 (TIANGEN, CN). The transformed strain was selected on LB plates containing kanamycin (30ug/mL). The pMO13: *orpD* plasmid was transferred to the HV020 using electricity strain and plated onto LB agar containing tellurite (30ug/mL). Complementation was confirmed by PCR using the primers described above based on product size and DNA sequencing.

### Statistical analyses

Statistical analyses were performed using GraphPad Prism (version 8.0) (GraphPad Software, Inc.), applying the student’s t-test, one-way ANOVA, followed by the Bonferroni post-hoc test for multiple comparisons to compare the survival rates of patients and mice in different groups. In mouse kill-assay, log-rank (Mantel-Cox) test was applied. The p values <0.05 were considered significant.

### Ethics statement

The mice were bred and maintained in specific pathogen-free animal facilities, and all procedures involving animals were approved by the Animal Ethics Committee.

## Results

### *Isolation and characterization of carbapenem-resistant* A. baumannii

A total of 70 inpatients from ICU, neurology department, infectious department, etc. in Beijing Ditan hospital were enrolled in this study, and 70 non-repetitive CRAB isolates were isolated from these patients. An environmental strain from a stethoscope was also collected from the ICU. Most of the isolates from patients were obtained from the respiratory tract (n = 54, 77.14%). Among the 70 patients, 56 (80%) were admitted to the ICU, 66 (89.19%) had pulmonary infections, and 36 (51.43%) developed respiratory failure. All patients were treated with no fewer than two antibiotics within 30 days, and 32 (45.71%) were treated with carbapenems.

**Figure 1.**
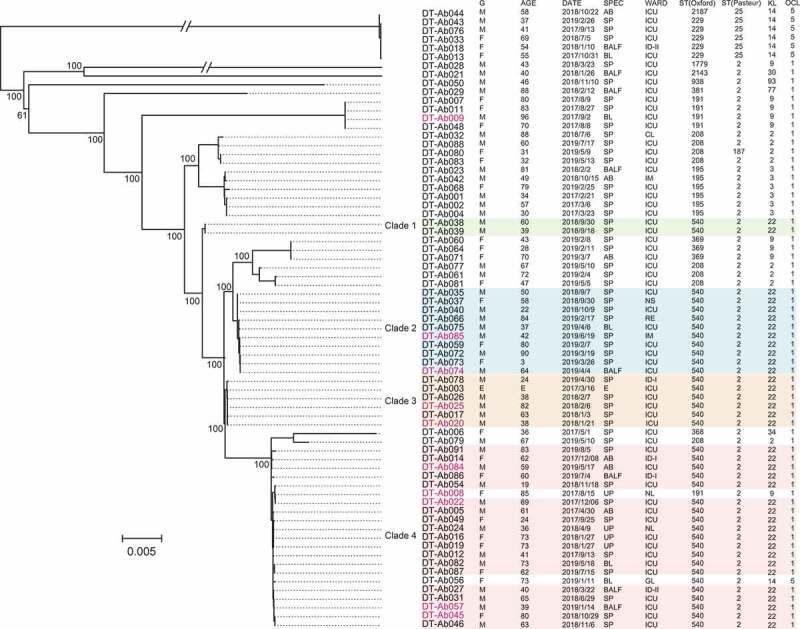
Phylogenetic relationships of 71 CRAB isolates based on core genome SNPs. The green, blue, orange and pink shading indicates the 4 ST2/KL22 clades respectively, the red characters denote the 10 deaths. Information about the strain ID, patient gender (g), patient age (AGE), date of isolation (DATE), specimen type (SPEC), inpatient ward (WARD), and sequence type of the strain (ST) are listed at the right of the phylogenetic tree. Pink markers show isolates from patients who died. Intensive Care Unit (ICU); Cardiovascular Department (CL); Integrated TCM & Western Medicine Department (IM); Respiration Department (RE); Disease Department (ID); Department of Gynecology (GL), Neurology (NL), Neurosurgery (NS). Sputum (SP); Ascites (AB); Urine (UP); Blood (BL); Bronchoalveolar lavage fluid (BALF); Environment (e). the sequence type, capsular polysaccharide (KL) and the lipooligosaccharide outer core were listed in the figure

We identified the sequence types of the isolates based on two widely used MLST schemes. According to the Pasteur scheme, the 71 isolates were assigned to ST2 (64 isolates), ST25 (6 isolates), and ST187 (1 isolate). Based on the Oxford scheme, the isolates were assigned to 10 STs, including ST540 (37 isolates), ST208 (8 isolates), ST195 (6 isolates), ST191 (5 isolates), and ST229 (5 isolates), etc. The five ST229 isolates and an ST2187 isolate of Oxford scheme belonged to ST25 (Pasteur), and isolates of other STs of Oxford scheme belonged to ST2 (Pasteur), except that strain DT-Ab080 belonged to ST187 (Pasteur), which is single locus variant of ST2. The DT-Ab080 was clustered in the same clonal complex, CC2 (Pasteur) as the ST2 strains. In addition, based on the identification of capsular polysaccharide and lipooligosaccharide outer core synthesis genes, all of the ST540 (Oxford) isolates belonged to ST2 (Pasteur)/KL22/OCL1 and all non-ST540 (Oxford) isolates belonged to different KL types (Figure 1).

### The long-standing ST2/KL22 is associated with severe outcomes

To illustrate the genomic characteristics and phylogenetic relationships of these CRAB strains, we performed whole genome sequencing on the 71 strains and obtained 1.42 ± 0.22 Gb clear read data per strain on average. Based on the SNP analysis, we obtained 32,769 cgSNPs. The phylogenetic tree based on these cgSNP sites revealed that the CRAB population consisted of two dominant branches, in which the ST25 (Pasteur) isolates separated from the other ST2/ST187 isolates (Figure 1). Due to genetic differences in strains of the same ST, the ST2/KL22 isolates were divided into four clades. Clade 1 included two isolates with one SNP, which were collected from two patients in the ICU in September 2019. Clade 2 included eleven isolates collected from patients in four departments within six months (September 2018 to April 2019), with an average of 6 ± 3.82 SNPs. Clade 3 had five isolates collected from patients between January 2018 and February 2018. The isolate DT-Ab003, obtained from a stethoscope in the ICU in March 2017, belonged to clade 3. The average number of SNPs in the clade 3 strains was 2 ± 1.47. Clade 4 included 19 strains isolated between April 2017 and July 2019 and caused five death events (Figure 1). The strains in clade 4 had an average of 15 ± 8.83 SNPs, indicating that they were derived from a long-standing clone in the hospital (Table S2).

**Figure 2.**
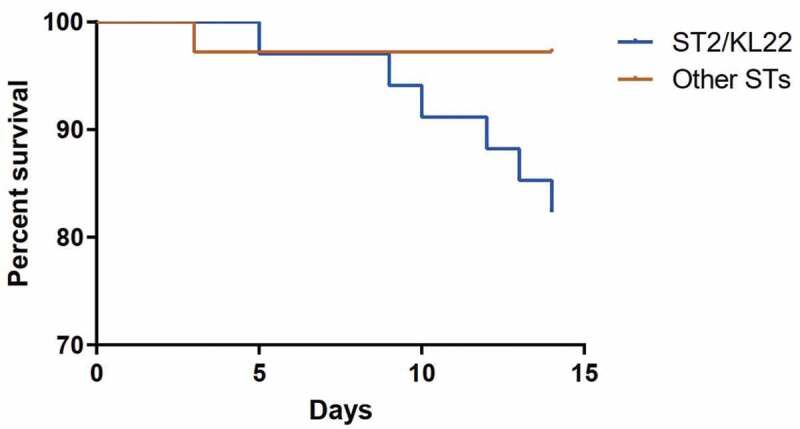
Survival rates of patients in the ST2/KL22 group and non- ST2/KL22 group over 14 days. The mortality rate of patients infected by the ST2/KL22 clone was higher than that of patients infected with other clones (*p = 0.04*)

In total, 37 of 71 isolates (52.11%) belonged to ST2/KL22. The 14-day mortality of patients infected with ST2/KL22 was significantly higher than that of the patients infected with other isolates (Figure 2). We then analyzed the clinical characteristics of patients infected with *A. baumannii* in the ST2/KL22 group and compared them with patients infected with non-ST2/KL22 group strains. Procalcitonin levels were significantly higher in patients infected with strains from the ST2/KL22 group (*p = 0.04*), indicating more severe inflammation and infection in these patients. There were no significant differences between the two groups in terms of number of surgeries, pulmonary infection, abdominal infection, co-morbidities, antibiotic selection, and laboratory results (C-reactive protein level and white blood cell count), but there was a significant difference in gender (*p = 0.02*) (Table 1).

**Table 1.** Demographic and Clinical Characteristics of 70 Patients with CRAB Infection Enrolled in this Study

	ST540 (n = 34)	Non-ST540 (n = 36)	P
Age (years), mean ± SD	53.56 ± 21.65	59.00 ± 19.48	0.48
Male gender	25	17	0.02*
Comorbid disease			
Pulmonary infection	16	15	0.65
Abdominal infection	6	6	0.91
Chronic kidney disease	3	5	0.51
Chronic liver failure	4	3	0.63
Coronary disease	5	2	0.20
COPD	1	1	0.97
Diabetes mellitus	7	7	0.90
Neurological disease	12	10	0.50
Cancer	4	4	0.93
Organ failure			
Respiratory failure	19	17	0.47
Shock	6	10	0.24
Laboratory finding			
WBC count, mean ± SD	9.09 ± 3.82	10.79 ± 6.54	0.20
PCT, mean ± SD	4.87 ± 14.19	3.33 ± 5.50	0.04*
CRP level, mean ± SD	99.65 ± 127.60	72.98 ± 65.70	0.77
Infectious disease			
HIV	6	3	0.24
Syphilis	0	2	0.16
Hepatitis B virus	0	1	0.33
Epstein-Barr virus infection	1	0	0.30
Invasive procedures			
Mechanical ventilation	20	21	0.97
Central venous catheter	22	23	0.94
Foley catheter	31	32	0.75
Use of antibiotics within 30 days prior to bacteremia			
Lactamase Inhibitors	12	17	0.31
Carbapenems	15	17	0.79
Quinolones	6	9	0.38
Cephalosporin	4	2	0.35
Aminoglycoside	3	5	0.51
Glycopeptide	6	12	0.13
Antifungal	9	11	0.71
7-day mortality	1	1	0.97
14-day mortality	6	1	0.04*
28-day mortality	7	2	0.06

Abbreviations: COPD, chronic obstructive pulmonary disease; WBC, white blood cell; PCT, procalcitonin; CRP, C-reactive protein; HIV, human immunodeficiency virus continuous renal replacement therapy; In total, 70 patients were included for the analysis of 7-day, 14-day and 28-day mortality. *P < 0.05: ST540 group vs non-ST540 group (14-day mortality; P = 0.04; pairwise analysis).

### *Carriage of reported virulence genes in the carbapenem-resistant* A. baumannii *isolates*

**Figure 3.**
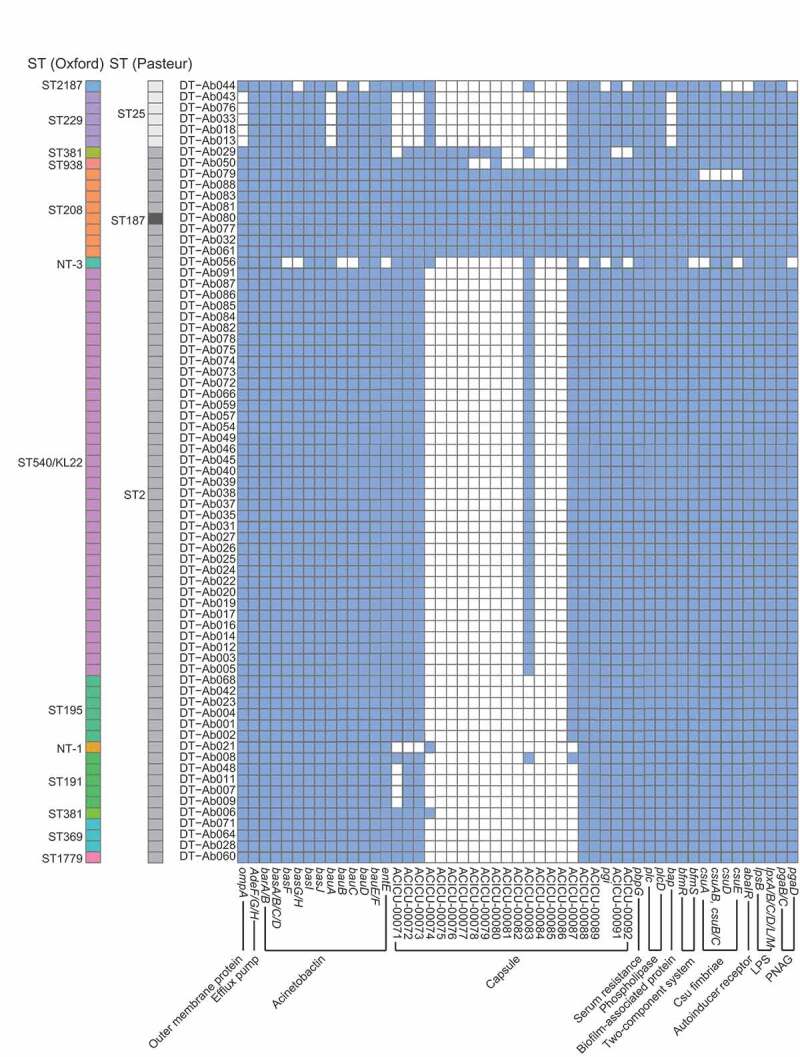
Distribution of virulence genes in the 71 CRAB isolates. The *bap, csu* and *qsar* genes are associated with biofilm formation. The *phospholipase, pbpg*, and *capsule* genes as well as the *bas-bau-bar* gene cluster are related to bacteria survival in human serum. The *lps* and *ompa* genes are related to cell invasion and apoptosis of the host cell. A blue box indicates that a strain (Y axis) contains a specific virulence factor (X axis), and white represents a negative gene alignment result. The sequence type (Oxford and Pasteur) of the strains was labeled

We analyzed the virulence factors in the isolates according to the comparison with *A. baumannii* virulence genes recorded in VFDB. Genes encoding twelve groups of common virulence factors in *A. baumannii* were identified **(Table S3)**. The septic shock-related gene *lps* (involved in lipid A biosynthesis) was identified in all CRAB isolates. The presence of biofilm formation-associated genes *bap* encoding BAPs, *csu* (Csu pili), and *qsar* (Quorum Sensing Autoinducer Receptor) were assessed, but there were no differences between the two groups. The ST2/KL22 isolates carried a higher number of genes than non-ST2/KL22 isolates related to bacterial survival in serum, including the gene cluster encoding Acinetobactin (*bas-bau-bar*). The gene encoding Outer membrane protein A (*omp A*), associated with epithelial cell invasion and apoptosis, was not detected in all of the five ST229 isolates (Figure 3, Table S4). In addition to the carriage of these virulence genes, we also identified the subtypes of BAPs and BLPs which were associated with biofilm formation and adhesion to host cells. In total, 69 out of the 71 isolates encoded type 2 or type 3 BAP, and only two isolates (DTAb044 and DTAb056) encoded both type 2 and type 3 BAPs. All ST2 (Pasteur) isolates encoded both type 1 and type 2A BLPs, and ST25 isolates encoded type 2A BLP except DTAb044 that encoded type 1 BLP. Although the inflammatory status (procalcitonin level) and outcomes were more severe in the patients infected with the ST2/KL22 strains, we did not find any significant different carriages of known virulence genes compared to non-ST2/KL22 isolates.

### A new OprD/Occ protein associated with the pathogenicity of ST2/KL22

We then deep analyzed the genomes of ST2/KL22 isolates based on the core and pan-genome analysis to explore more factors associated with the pathogenicity. We identified six specific genes in long-standing clade 4 clone (from December 2017 to July 2019), which are located within a ~ 10.8 kb fragment (Table S5). Among them, a gene encoding an OprD family protein was identified as a virulence associated by gene annotation and literature study. This gene family encodes outer-membrane porins involved in bacterial survival and virulence in the host [29]. Based on sequence comparison and phylogenetic analysis, the OprD specific in the long-standing clade 4 clone was not clustered with those in Pseudomonas aeruginosa and occAB1, occAB2, occAB3 and occAB4 identified in *A. baumannii* previously, indicating a new OprD/Occ subgroup **(Figure S1, red triangle)**.

**Figure 4.**
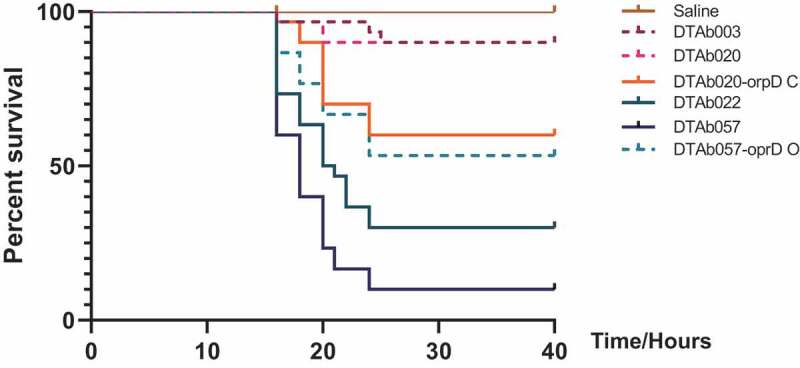
Virulence potential of ST2/KL22 CRAB isolates. The virulence of two isolates from clade 3 (DT-Ab003: purple and DT-Ab020: pink) and two from clade 4 (DT-Ab022: cyan and DT-Ab057: blue) in the C57 mouse infection model. The dark green represents saline, and the orange referred to the DT-Ab020 expressed a complete *oprD* gene cloned from DT-Ab057

The pathogenicity of two clade 4 strains (DT-Ab022 and DT-Ab057) were evaluated by performing *in vivo* virulence experiments in intraperitoneal injection mouse model. The control strains (lacking *oprD* but carrying all other virulence genes), DT-Ab003, and DT-Ab020, were selected from clade 3, which is most closely related to clade 4. C57 mice were infected with the four isolates by intraperitoneal injection (2 × 10^8^ c.f.u. each), and the survival rate at 40 h post-infection was calculated. The survival rates of C57 mice were 90% (DT-Ab003), 90% (DT-Ab020), 30% (DT-Ab022), and 10% (DT-Ab057) at 40 h (Figure 4, Table S6). These results were in line with the clinical data, both of which revealed that clade 4 was a high-virulent clone.

### *Validation of the* oprD *gene contributing to the virulence of ST2/KL22 strains by genome comparison, gene knock out and complement*

**Figure 5.**
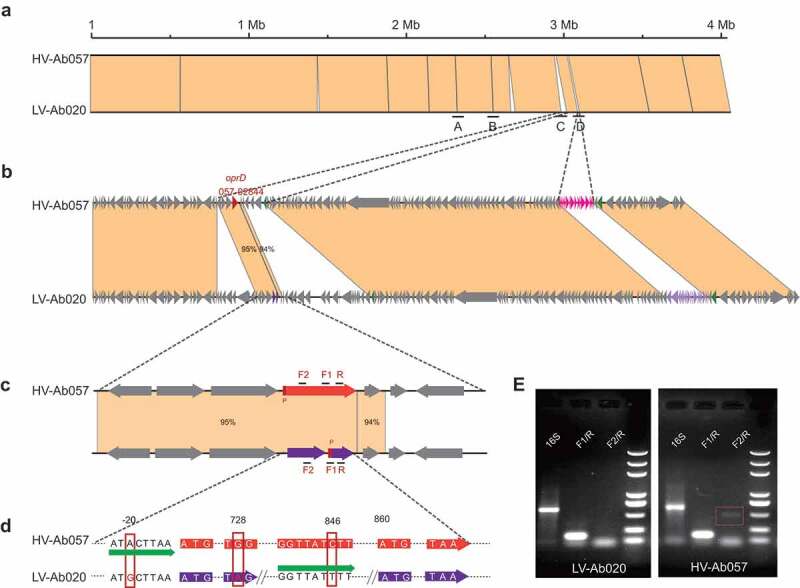
Structure and expression analysis of the *oprD* in LV and HV strains. A. Comparison of four loci (A, B, C and D) that differ between HV-Ab057and LV-Ab020. B. The different sequences in Locus C and Locus D. C. Locus C in HV-Ab057 (orange) and LV-Ab020 (purple). D. Genetic mutations in HV-Ab057 and LV-Ab020. E. Expression of the wild-type and mutant *orpD* gene in HV-Ab057 (orange) and the mutant gene LV-Ab020, respectively

To confirm the contribution of the *oprD* gene in the pathogenicity of ST540 isolates, we first compared the complete genome sequences of high (or relatively high) virulence strain: DT-Ab022 (High Virulence Ab022, HV-Ab022), DT-Ab057 (High Virulence Ab057, HV-Ab057), and low-virulence strains DT-Ab003 (Low Virulence Ab003, LV-Ab003), DT-Ab020 (Low Virulence Ab020, LV-Ab020). A total of four different loci (A, B, C, and D) that differed between the HV and LV strains were identified (Figure 5a). Loci A and B were identified as intergenic regions that are predicted to have negligible effects on protein coding genes and bacterial virulence. In addition, sequence analysis revealed that two distinct prophage-like gene clusters were inserted in the loci D of two genomes. Gene function analysis showed no virulence-related genes in the two prophage-like gene clusters **(Table S7)**. In the HV-Ab057, a 1,347 bp open reading frame (ORF) of *orpD* (DT-Ab057_02847) and its cis-regulatory element (P-*orpD*) were detected in loci C Figure 5b, c, Table S7). By contrast, in LV-Ab020 two ORFs were predicted in the corresponding region. On the 243th amino acid of *oprD*, a SNP of G->A caused mutation of TGG (tryptophan) to TAG (stop codon), leading to a premature termination codon, and then the loci were separated into two ORFs (DT-Ab020_02900 and DT-Ab020_02901). In addition, P-*orpD* was lost by a point mutation, and another promoter was identified upstream of DT-Ab020_02901 (Figure 5d).

Next, we detected the expression of the *oprD* gene in HV-Ab057 and LV-Ab020. Reverse-transcription PCR analysis of gene expression revealed that the 3ʹ part of the *oprD* transcript could be amplified from both HV-Ab057and LV-Ab020, when using the primer pair F1/R. By contrast, after amplification with the primers F2/R, which are designed to amplify the full-length *oprD* transcript, products were only obtained from HV-Ab057, indicating that the transcript was truncated in LV-Ab020 (Figure 5e, Figure S2). Finally, we validated the function of the *oprD* gene by constructing gene knock out and complement strains, and then performing *in vivo* virulence experiments. An HV-057 mutant knocking out *oprD* and an LV-020 containing a complete *orpD* gene on an expression plasmid (pMO13: *orpD*) were constructed. By the survival experiment with C57 model, the virulence was reduced in the *oprD* knockout HV-057 mutant, with an increased survival rate from 10% to 50%. In contrast, the virulence was enhanced in the *oprD* complemented LV-020 mutant, such that the survival rate was reduced from 90% to 60% (Figure 4, table S6).

## Discussion

The emerging and spread of multi-drug resistant *A. baumannii* in hospitals worldwide has become a serious public health threat [30]. Few antibiotics are effective for treating infections caused by this pathogen. To date, several major epidemic lineages, or international clones, have been reported globally and caused a broad range of severe nosocomial infections. To overcome this problem, knowledge of the epidemic characteristics, pathogenesis, and antibiotic resistance mechanisms of *A. baumannii* is important [31]. Large amount of studies had been performed to uncover the molecular characteristics, resistance profiles, and mechanisms of this pathogen. In this study, ST2/KL22 was found as the dominant ST in the hospital, which was also reported as one of the dominant lineages worldwide. Moreover, ST2/KL22 was found to cause higher mortality rates. Based on whole-genome sequencing and virulence experiments, we confirmed a long-standing and virulence-enhanced ST2/KL22 clone in our hospital.

We identified a novel porin gene, *oprD*, that contributed to the high virulence of ST2/KL22 clone by genomic comparison between high-virulent and low virulent isolates firstly. Porins are outer membrane proteins associated with modulating cellular permeability. In many gram-negative bacteria, OrpD porin is involved in carbapenem resistance, but the antibiotic effect of several antibiotics (chloramphenicol, aztreonam, and nalidixic acid) in the isolates with or without the complete *orpD* gene was not affected [32]. In several gram-negative bacteria like *P. aeruginosa*, OrpD also contributes to adaptation to specific environments including the maintenance of *P. aeruginosa* biofilms [33]. Furthermore, OprD had functions in adhesion, signaling, the diffusion of nutrients and the uptake of gluconate [34]. However, to our knowledge, there were few studies identified or explored its role in the virulence of *A. baumannii*.

OmpA was the porin that proved to enhance the survival and persistence of *A. baumannii* by facilitating surface motility and biofilm formation *in vivo* [35]. OprD was a new porin defined in *A. baumannii*, which showed low with the reported oprD gene in *P.aeruginosa* and *A. baumannii*. The expression of OprD was in HV and LV CRAB isolates was analyzed through quantitative RT-PCR, and mechanism responsible for increase of virulence need to be further analyzed [36]. The complement of oprD strain did enhance the virulence of the LV-020, but the pathogenicity of mutant strain was not as strong as the HV-057 strain. A similar result was observed in the gene knock HV-057 strain, which indicates the explicit mechanism of OprD in the *A. baumannii* virulence remains to be determined by in-depth *in vivo* and *in vivo* analysis.

In addition to the *oprD* gene, there are other three genes specifically in Clade 4 within the ~10.8 kb fragment including *oprD*, which might also encode membrane proteins (Table S5). These proteins might be associated with the function of OprD and need further studies. Our results also revealed that the OprD porin can become novel treatment targets to deal with the multi-drug resistant *A. baumannii* due to its cell-member distribution characteristics. The molecules which could block OprD, such as the antibody, or the gene editing therapy like CRISPR system targeting *oprD*, might reduce the virulence of *A. baumannii* specifically with few effects on the normal flora. The new therapy alternative to antibiotics can meet the challenge of highly virulence-multidrug-resistant infections.

In conclusion, we have validated a new virulence-associate porin: OprD in *A. baumannii*, a superbug in hospital and particularly in ICU. The pathogenic mechanism of OprD might be different from OmpA, which needs further study. This gene and the OprD protein might also be specific treating targets of multidrug-resistant *A. baumannii*, which could be meaningful in the critical care medicine.

## Supplementary Material

Supplemental Material

Supplemental Material

Supplemental Material

Supplemental Material

Supplemental Material

Supplemental Material

Supplemental Material

Supplemental Material
