# Supplementary material for: The novel outer membrane protein from OprD/Occ family is associated with hypervirulence of carbapenem resistant Acinetobacter baumannii ST2/KL22
Source: Virulence. 2020 Dec 29;12(1):1–11. doi: 10.1080/21505594.2020.1856560 (PMC7781578; doi:10.1080/21505594.2020.1856560)
Supplement: Supplemental Material [file KVIR_A_1856560_SM8669.docx]

| **Different Loci** | **Location on DT-Ab057** | **Genome content on DT-Ab057** | **Location on DT-Ab020** | **Genome content on DT-Ab020** |
| --- | --- | --- | --- | --- |
| Locus A | 2318881 | intergenic | 2330064-2331245 | ISAba1,ISAba33 |
| Locus B | 2546092 | intergenic | 2558465-2559646 | ISAba1,ISAba33 |
| Locus C | 2947961-2958852 | DT-Ab057_02841-DT-Ab057_02852 | 2989947-3026493 | DT-Ab020_02888-DT-Ab020_02921 |
| Locus D | 3028943-3037493 | DT-Ab057_02925-DT-Ab057_02941 | 3096583-3106653 | DT-Ab020_02994-DT-Ab020_03011 |

**Table S7. Different gene Loci between low and high virulence ST2/KL22 strains.**

**Different genes in Locus C**

| **Genome content** | **Description** | **Genome content** | **Description** |
| --- | --- | --- | --- |
| DT-Ab057_02844 | Transcriptional regulator | DT-Ab020_02888 | DUF454 domain-containing protein |
| DT-Ab057_02845 | Dihydrodipicolinate synthase family protein | DT-Ab020_02889 | Heme oxygenase |
| DT-Ab057_02846 | MFS transporter | DT-Ab020_02890 | Energy transducer TonB |
| **DT-Ab057_02847** | **Outer membrane porin, OprD family** | DT-Ab020_02891 | Peptide signal protein |
| DT-Ab057_02849 | Putative membrane protein | DT-Ab020_02892 | Transferrin-binding protein-like solute binding protein |
| DT-Ab057_02850 | Putative membrane protein | DT-Ab020_02893 | Outer membrane receptor |
| DT-Ab057_02851 | DNA adenine methylase | DT-Ab020_02894 | FecR family protein |
| DT-Ab057_02852 | ATP-binding protein | DT-Ab020_02895 | RNA polymerase subunit |
|  |  | DT-Ab020_02896 | LysR family transcriptional regulator |
|  |  | DT-Ab020_02897 | Dihydrodipicolinate synthase family protein |
|  |  | DT-Ab020_02898 | MFS transporter |
|  |  | **DT-Ab020_02899** | **Outer membrane porin, OprD family** |
|  |  | **DT-Ab020_02900** | **Outer membrane porin, OprD family** |
|  |  | DT-Ab020_02902 | Integron gene cassette protein |
|  |  | DT-Ab020_02903 | TatD family hydrolase |
|  |  | DT-Ab020_02906 | KAP family P-loop domain protein |
|  |  | DT-Ab020_02907 | Competence protein |
|  |  | DT-Ab020_02909 | Nucleoid-associated protein |
|  |  | DT-Ab020_02910 | Single-stranded DNA-binding protein |
|  |  | DT-Ab020_02911 | Single-stranded DNA-binding protein |
|  |  | DT-Ab020_02912 | Inner membrane protein |
|  |  | DT-Ab020_02917 | gp54 protein |
|  |  | DT-Ab020_02918 | DNA-binding protein |
|  |  | DT-Ab020_02919 | Transcriptional regulator, partial |
|  |  | DT-Ab020_02921 | Site-specific recombinase, phage integrase family |

**Different genes in Locus D**

| **Genome content** | **Description** | **Genome content** | **Description** |
| --- | --- | --- | --- |
| DT-Ab057_02925 | DUF1376 domain-containing protein | DT-Ab020_02994 | DUF1376 domain-containing protein |
| DT-Ab057_02928 | Putative uncharacterized protein | DT-Ab020_02995 | Site-specific DNA methylase |
| DT-Ab057_02929 | Bacteriophage CII protein | DT-Ab020_02996 | Site-specific DNA methylase |
| DT-Ab057_02931 | Phage repressor protein | DT-Ab020_03002 | Helix-turn-helix transcriptional regulator |
| DT-Ab057_02932 | LexA family transcriptional regulator | DT-Ab020_03003 | Helix-turn-helix transcriptional regulator |
| DT-Ab057_02934 | Putative membrane protein, partial | DT-Ab020_03005 | Prophage PssSM-02 |
| DT-Ab057_02935 | Putative membrane protein, partial | DT-Ab020_03006 | DUF2303 family protein |
| DT-Ab057_02937 | ATP-binding protein | DT-Ab020_03010 | DNA polymerase III subunit epsilon |
| DT-Ab057_02940 | DUF551 domain-containing protein | DT-Ab020_03011 | Ead/Ea22-like family protein |
