# Supplementary material for: The novel outer membrane protein from OprD/Occ family is associated with hypervirulence of carbapenem resistant Acinetobacter baumannii ST2/KL22
Source: Virulence. 2020 Dec 29;12(1):1–11. doi: 10.1080/21505594.2020.1856560 (PMC7781578; doi:10.1080/21505594.2020.1856560)
Supplement: Supplemental Material [file KVIR_A_1856560_SM8500.docx]

**Table S6**

1. **Survival proportions of all the groups**

| **Time(Hours)** | **PBS** | **DTAb003** | **DTAb020** | **DTAb022** | **DTAb020+orpD** | **DTAb057** | **DTAb057-oprD** |
| --- | --- | --- | --- | --- | --- | --- | --- |
| 0 | 100.00 | 100 | 100.00 | 100 | 100.00 | 100.00 | 100.00 |
| 16 | 100.00 | 96.67 | 96.67 | 73.33 | 96.67 | 60.00 | 86.67 |
| 18 |  |  |  | 63.33 | 90.00 | 40.00 | 76.67 |
| 20 |  |  | 90.00 | 50.00 | 70.00 | 23.33 | 66.67 |
| 21 |  |  |  | 46.67 |  | 16.67 |  |
| 22 |  |  |  | 36.67 |  |  |  |
| 24 |  | 93.33 |  | 30.00 | 60.00 | 10.00 | 53.33 |
| 25 |  | 90 |  |  |  |  |  |
| 40 | 100.00 | 90 | 90.00 | 30 | 60.00 | 10.00 | 53.33 |

1. **Survival Curve compraisons of all the groups**

| **Log-rank (Mantel-Cox) test (conservative)** |  |
| --- | --- |
| Chi square | 96.67 |
| df | 6 |
| P value | <0.0001 |
| P value summary | **** |
| Are the survival curves sig different? | Yes |

1. **Survival Curve compraisons of low- and high- virulence strains.**

| **Log-rank (Mantel-Cox) test (conservative)** |  |
| --- | --- |
| Chi square | 42.08 |
| df | 1 |
| P value | <0.0001 |
| P value summary | **** |
| Are the survival curves sig different? | Yes |

1. **Survival Curve compraisons of low-virulence and its oprD knock-in strains**

| **Log-rank (Mantel-Cox) test (conservative)** |  |
| --- | --- |
| Chi square | 6.797 |
| df | 1 |
| P value | 0.0091 |
| P value summary | ** |
| Are the survival curves sig different? | Yes |

1. **Survival Curve compraisons of high- virulence and its oprD knock-out strains.**

| **Log-rank (Mantel-Cox) test (conservative)** |  |
| --- | --- |
| Chi square | 16.11 |
| df | 1 |
| P value | <0.0001 |
| P value summary | **** |
| Are the survival curves sig different? | Yes |
