# Supplementary material for: The novel outer membrane protein from OprD/Occ family is associated with hypervirulence of carbapenem resistant Acinetobacter baumannii ST2/KL22
Source: Virulence. 2020 Dec 29;12(1):1–11. doi: 10.1080/21505594.2020.1856560 (PMC7781578; doi:10.1080/21505594.2020.1856560)
Supplement: Supplemental Material [file KVIR_A_1856560_SM8472.docx]

**Table S5. Specific genes in long-standing clade 4 strains**

| **Gene** | **Clade 1 (n=2)** | **Clade 2 (n=10)** | **Clade 3 (n=6)** | **Clade 4 (n=19)** | **Annotation(Protein)** |
| --- | --- | --- | --- | --- | --- |
| *DT-Ab057_02843* | 0 | 0 | 0 | 19 | Hypothetical Protein |
| *DT-Ab057_02847* | 0 | 0 | 0 | 19 | OprD |
| *DT-Ab057_02849* | 0 | 0 | 1 | 19 | Putative Membrane Protein |
| *DT-Ab057_02850* | 0 | 0 | 0 | 19 | Membrane Protein |
| *DT-Ab057_02851* | 0 | 0 | 0 | 19 | DNA Methyltransferase |
| *DT-Ab057_02852* | 0 | 0 | 0 | 19 | Peptide ABC Transporter ATP-Binding Protein |
