# Supplementary material for: The novel outer membrane protein from OprD/Occ family is associated with hypervirulence of carbapenem resistant Acinetobacter baumannii ST2/KL22
Source: Virulence. 2020 Dec 29;12(1):1–11. doi: 10.1080/21505594.2020.1856560 (PMC7781578; doi:10.1080/21505594.2020.1856560)
Supplement: Supplemental Material [file KVIR_A_1856560_SM8462.docx]

**Table S4. Distribution of virulence genes in the ST2/KL22 group and non- ST2/KL22 group**

| **Function in pathogenesis** | **Virulence gene** | | **ST2/KL22** | | **non- ST2/KL22** | | **P** |
| --- | --- | --- | --- | --- | --- | --- | --- |
| Biofilm formation and  bacteria survival | *bap* |  | 34 | 100.00% | 26 | 72.22% | **0.0009** |
|  | *bfmrs.bfmr* | | 34 | 100.00% | 36 | 100.00% | >0.9999 |
|  | *bfmrs.bfms* | | 34 | 100.00% | 36 | 100.00% | >0.9999 |
|  | *pnag.pgad* | | 34 | 100.00% | 36 | 100.00% | >0.9999 |
|  | *pnag.pgab.c* | | 34 | 100.00% | 36 | 100.00% | >0.9999 |
|  | *csu_fimbriae.csue* | | 34 | 100.00% | 35 | 97.22% | 0.3277 |
|  | *csu_fimbriae.csua* | | 34 | 100.00% | 35 | 97.22% | 0.3277 |
|  | *csu_fimbriae.csub* | | 34 | 100.00% | 35 | 97.22% | 0.3277 |
|  | *csu_fimbriae.csua.b* | | 34 | 100.00% | 35 | 97.22% | 0.3277 |
|  | *csu_fimbriae.csuc* | | 34 | 100.00% | 35 | 97.22% | 0.3277 |
|  | *csu_fimbriae.csud* | | 34 | 100.00% | 33 | 91.67% | **0.0853** |
|  | *quorom_sensing.autoinducer.receptor* | | 34 | 100.00% | 34 | 94.44% | 0.1632 |
| Survival in human serum | *acinetobactin.baua* | | 34 | 100.00% | 30 | 83.33% | **0.0128** |
|  | *acinetobactin.basa* | | 34 | 100.00% | 30 | 83.33% | **0.0128** |
|  | *acinetobactin.bara.b* | | 34 | 100.00% | 31 | 86.11% | **0.0241** |
|  | *acinetobactin.baue.f* | | 34 | 100.00% | 31 | 86.11% | **0.0241** |
|  | *acinetobactin.ente* | | 34 | 100.00% | 31 | 86.11% | **0.0241** |
|  | *acinetobactin.bauc* | | 30 | 88.24% | 25 | 69.44% | 0.0555 |
|  | *acinetobactin.baub* | | 34 | 100.00% | 36 | 100.00% | >0.9999 |
|  | *acinetobactin.baud* | | 34 | 100.00% | 36 | 100.00% | >0.9999 |
|  | *phospholipase* | | 34 | 100.00% | 36 | 100.00% | >0.9999 |
|  | *pbpg* | | 34 | 100.00% | 36 | 100.00% | >0.9999 |
|  | *capsule00087* | | 34 | 100.00% | 35 | 97.22% | 0.3277 |
|  | *capsule00083* | | 34 | 100.00% | 35 | 97.22% | 0.3277 |
|  | *capsule00073* | | 34 | 100.00% | 35 | 97.22% | 0.3277 |
|  | *capsule00072* | | 34 | 100.00% | 34 | 94.44% | 0.1632 |
|  | *capsule.acicu_00092* | | 34 | 100.00% | 34 | 94.44% | 0.1632 |
|  | *capsule.acicu_00089* | | 34 | 100.00% | 34 | 94.44% | 0.1632 |
|  | *capsule.acicu_00091* | | 34 | 100.00% | 33 | 91.67% | 0.0853 |
|  | *capsule.acicu_00088* | | 34 | 100.00% | 35 | 97.22% | 0.3277 |
|  | *capsule.acicu_00071* | | 34 | 100.00% | 35 | 97.22% | 0.3277 |
|  | *capsule.acicu_00079* | | 34 | 100.00% | 34 | 94.44% | 0.1632 |
|  | *capsule.acicu_00078* | | 0 | 0.00% | 9 | 25.00% | **0.0018** |
|  | *capsule.acicu_00076* | | 0 | 0.00% | 9 | 25.00% | **0.0018** |
|  | *capsule.acicu_00075* | | 0 | 0.00% | 10 | 27.78% | **0.0009** |
|  | *capsule.acicu_00077* | | 0 | 0.00% | 10 | 27.78% | **0.0009** |
|  | *capsule.acicu_00080* | | 0 | 0.00% | 10 | 27.78% | **0.0009** |
|  | *capsule.acicu_00082* | | 0 | 0.00% | 10 | 27.78% | **0.0009** |
|  | *capsule.acicu_00081* | | 0 | 0.00% | 8 | 22.22% | **0.0035** |
|  | *capsule.acicu_00084* | | 0 | 0.00% | 8 | 22.22% | **0.0035** |
|  | *capsule.acicu_00085* | | 0 | 0.00% | 8 | 22.22% | **0.0035** |
|  | *capsule.acicu_00086* | | 0 | 0.00% | 8 | 22.22% | **0.0035** |
|  | *capsule.acicu_00074* | | 0 | 0.00% | 8 | 22.22% | **0.0035** |
|  | *capsule.pgi* | | 1 | 2.94% | 18 | 50.00% | **<0.0001** |
| **Cell invasion and apoptosis** | ***ompA*** | | **34** | **100.00%** | **14** | **38.89%** | **<0.0001** |
| Increased antibiotic tolerance | *adefgh_efflux_pump.adef.g.h* | | 34 | 100.00% | 36 | 100.00% | >0.9999 |
| Septic shock | *lps.lpsb* | | 34 | 100.00% | 36 | 100.00% | >0.9999 |
|  | *lps.lpx* | | 34 | 100.00% | 36 | 100.00% | >0.9999 |
