# Supplementary material for: The novel outer membrane protein from OprD/Occ family is associated with hypervirulence of carbapenem resistant Acinetobacter baumannii ST2/KL22
Source: Virulence. 2020 Dec 29;12(1):1–11. doi: 10.1080/21505594.2020.1856560 (PMC7781578; doi:10.1080/21505594.2020.1856560)
Supplement: Supplemental Material [file KVIR_A_1856560_SM8452.docx]

**Table S3. The functions of virluence genes in *A. baumannii***

| **Virulence gene** | **Abbreviation** | **Function in pathogenesis** |
| --- | --- | --- |
| *penicillin-binding protein* | *pbpg* | Survival in human serum |
| *out member protein A* | *ompA* | Epithelial cell invasion and apoptosis |
| *biofilm-associated gene* | *bap* | Biofilm formation and help sustain cell survival on biotic and abiotic surfaces |
|  | *quorum-sensing system* | Biofilm formation and help sustain cell survival on biotic and abiotic surfaces |
|  | *phospholipases* | Survival in human serum and epithelial cell invasion |
| *β-(1-->6)-poly-n-acetyl-d-glucosamine* | *pnag* | Biofilm formation and help sustain cell survival on biotic and abiotic surfaces |
| *lipopolysaccharide* | *lps* | Causing septic shock |
|  | *csu* | Biofilm formation and help sustain cell survival on biotic and abiotic surfaces |
|  | *capsule* | Survival in human serum |
| *biofilm formation* | *bfmrs* | Biofilm formation and help sustain cell survival on biotic and abiotic surfaces |
|  | *adefgh efflux pumps* | Increased antibiotic tolerance |
|  | *acinetobactin* | Survival in human serum |
