# Supplementary material for: The novel outer membrane protein from OprD/Occ family is associated with hypervirulence of carbapenem resistant Acinetobacter baumannii ST2/KL22
Source: Virulence. 2020 Dec 29;12(1):1–11. doi: 10.1080/21505594.2020.1856560 (PMC7781578; doi:10.1080/21505594.2020.1856560)
Supplement: Supplemental Material [file KVIR_A_1856560_SM8450.docx]

|  | DT-Ab035 | DT-Ab066 | DT-Ab075 | DT-Ab085 | DT-Ab072 | DT-Ab059 |
| --- | --- | --- | --- | --- | --- | --- |
| DT-Ab035 | 0 | 7 | 9 | 10 | 11 | 11 |
| DT-Ab066 | 7 | 0 | 4 | 5 | 6 | 6 |
| DT-Ab075 | 9 | 4 | 0 | 7 | 8 | 8 |
| DT-Ab085 | 10 | 5 | 7 | 0 | 1 | 1 |
| DT-Ab072 | 11 | 6 | 8 | 1 | 0 | 2 |
| DT-Ab059 | 11 | 6 | 8 | 1 | 2 | 0 |

**Table S2.**

**a) Number of SNPs in ST2/KL22 clade 2 isolates**

Strains without SNP: DT-Ab035, DT-Ab037, and DT-Ab040; DT-Ab072, DT-Ab073, and DT-Ab074

**b) Number of SNPs in ST2/KL22 clade 3 isolates**

|  | DT-Ab003 | DT-Ab025 | DT-Ab020 | DT-Ab026 | DT-Ab017 |
| --- | --- | --- | --- | --- | --- |
| DT-Ab003 | 0 | 1 | 2 | 2 | 4 |
| DT-Ab025 | 1 | 0 | 1 | 1 | 3 |
| DT-Ab020 | 2 | 1 | 0 | 2 | 4 |
| DT-Ab026 | 2 | 1 | 2 | 0 | 4 |
| DT-Ab017 | 4 | 3 | 4 | 4 | 0 |

**c) Number of SNPs in ST ST2/KL22 clade 4 isolates**

|  | DT-Ab005 | DT-Ab016 | DT-Ab012 | DT-Ab024 | DT-Ab027 | DT-Ab031 | DT-Ab049 | DT-Ab082 | DT-Ab057 | DT-Ab045 | DT-Ab084 | DT-Ab014 | DT-Ab022 | DT-Ab054 | DT-Ab086 | DT-Ab091 |
| --- | --- | --- | --- | --- | --- | --- | --- | --- | --- | --- | --- | --- | --- | --- | --- | --- |
| DT-Ab005 | 0 | 6 | 6 | 7 | 7 | 8 | 9 | 9 | 10 | 12 | 14 | 17 | 17 | 19 | 22 | 29 |
| DT-Ab016 | 6 | 0 | 4 | 1 | 5 | 6 | 3 | 7 | 8 | 10 | 12 | 15 | 15 | 17 | 20 | 27 |
| DT-Ab012 | 6 | 4 | 0 | 5 | 5 | 6 | 7 | 7 | 8 | 10 | 12 | 15 | 15 | 17 | 20 | 27 |
| DT-Ab024 | 7 | 1 | 5 | 0 | 6 | 7 | 4 | 8 | 9 | 11 | 13 | 16 | 16 | 18 | 21 | 28 |
| DT-Ab027 | 7 | 5 | 5 | 6 | 0 | 3 | 8 | 8 | 5 | 7 | 13 | 16 | 16 | 18 | 21 | 28 |
| DT-Ab031 | 8 | 6 | 6 | 7 | 3 | 0 | 9 | 9 | 4 | 6 | 14 | 17 | 17 | 19 | 22 | 29 |
| DT-Ab049 | 9 | 3 | 7 | 4 | 8 | 9 | 0 | 10 | 11 | 13 | 15 | 18 | 18 | 20 | 23 | 30 |
| DT-Ab082 | 9 | 7 | 7 | 8 | 8 | 9 | 10 | 0 | 11 | 13 | 15 | 18 | 18 | 20 | 23 | 30 |
| DT-Ab057 | 10 | 8 | 8 | 9 | 5 | 4 | 11 | 11 | 0 | 8 | 16 | 19 | 19 | 21 | 24 | 31 |
| DT-Ab045 | 12 | 10 | 10 | 11 | 7 | 6 | 13 | 13 | 8 | 0 | 18 | 21 | 21 | 23 | 26 | 33 |
| DT-Ab084 | 14 | 12 | 12 | 13 | 13 | 14 | 15 | 15 | 16 | 18 | 0 | 7 | 19 | 21 | 14 | 19 |
| DT-Ab014 | 17 | 15 | 15 | 16 | 16 | 17 | 18 | 18 | 19 | 21 | 7 | 0 | 22 | 24 | 17 | 22 |
| DT-Ab022 | 17 | 15 | 15 | 16 | 16 | 17 | 18 | 18 | 19 | 21 | 19 | 22 | 0 | 4 | 27 | 34 |
| DT-Ab054 | 19 | 17 | 17 | 18 | 18 | 19 | 20 | 20 | 21 | 23 | 21 | 24 | 4 | 0 | 29 | 36 |
| DT-Ab086 | 22 | 20 | 20 | 21 | 21 | 22 | 23 | 23 | 24 | 26 | 14 | 17 | 27 | 29 | 0 | 29 |
| DT-Ab091 | 29 | 27 | 27 | 28 | 28 | 29 | 30 | 30 | 31 | 33 | 19 | 22 | 34 | 36 | 29 | 0 |

Strains without SNP: DT-Ab016 and DT-Ab019; DT-Ab045 and DT-Ab046; DT-Ab082 and DT-Ab087, respectively
