# Supplementary material for: The novel outer membrane protein from OprD/Occ family is associated with hypervirulence of carbapenem resistant Acinetobacter baumannii ST2/KL22
Source: Virulence. 2020 Dec 29;12(1):1–11. doi: 10.1080/21505594.2020.1856560 (PMC7781578; doi:10.1080/21505594.2020.1856560)
Supplement: Supplemental Material [file KVIR_A_1856560_SM8449.docx]

| **Name** | **Primer 5′-3′** |
| --- | --- |
| oprD-KO-UF | ttggtggtagGCCTCTCGGTTTTAGGCG |
| oprD-KO-UR | tttaaatatgGCCCTTTGCCTAACGCTG |
| oprD-KO-EF | ggcaaagggcCATATTTAAAAAGCTACCAAGAC |
| oprD-KO-ER | tagcttgtgcTTACTTATTAAATAATTTATAGCTATTGAAAAG |
| oprD-KO-DF | taataagtaaGCACAAGCTAAACGTTTAG |
| oprD-KO-DR | accgagaggcCTACCACCAAAGATCACC |
| oprD CF | cgtgctgacctgacctgagcCATCCATTTCATAAAAAGCTTG |
| oprD CR | ccgtcgcatgcatctagaggCGTTTAGCTTGTGCTTAAAATG |

**Table S1. Primers used in construction of *orpD* knock out and complement strains**
